# Successful extraction of insect DNA from recent copal inclusions: limits and perspectives

**DOI:** 10.1038/s41598-021-86058-9

**Published:** 2021-03-25

**Authors:** Alessandra Modi, Chiara Vergata, Cristina Zilli, Chiara Vischioni, Stefania Vai, Guidantonio Malagoli Tagliazucchi, Martina Lari, David Caramelli, Cristian Taccioli

**Affiliations:** 1 Department of Biology, University of Florence, 50122 Florence, Italy; 2 Independent Researcher, Padova, Italy; 3 Department of Animal Medicine, Production and Health, University of Padova, 35020 Legnaro, PD Italy; 4 UCL Genetics Institute, Department of Genetics, Evolution and Environment, University College London, Darwin Building, Gower Street, London, WC1E 6BT UK

**Keywords:** Biological techniques, Ecology, Molecular biology, Ecology, Environmental sciences

## Abstract

Insects entombed in copal, the sub-fossilized resin precursor of amber, represent a potential source of genetic data for extinct and extant, but endangered or elusive, species. Despite several studies demonstrated that it is not possible to recover endogenous DNA from insect inclusions, the preservation of biomolecules in fossilized resins samples is still under debate. In this study, we tested the possibility of obtaining endogenous ancient DNA (aDNA) molecules from insects preserved in copal, applying experimental protocols specifically designed for aDNA recovery. We were able to extract endogenous DNA molecules from one of the two samples analyzed, and to identify the taxonomic status of the specimen. Even if the sample was found well protected from external contaminants, the recovered DNA was low concentrated and extremely degraded, compared to the sample age. We conclude that it is possible to obtain genomic data from resin-entombed organisms, although we discourage aDNA analysis because of the destructive method of extraction protocols and the non-reproducibility of the results.

## Introduction

Since the first ancient DNA (aDNA) was successfully recovered from an extinct species in 1984^1^, palaeogenetic analyses have revolutionized many fields of research. In particular, bones and teeth have been the most used substrates over the last thirty years. However, current developments in Next Generation Sequencing (NGS) technologies coupled with optimized DNA extraction protocols^2–7^, have recently allowed to perform genomic analysis also using alternative biomaterials^8–14^. Nowadays, palaeogenetic researches involve historical and prehistoric findings hailing from archaeological excavations, libraries and museum collections. One of the alternative substrates from which scientists attempted to extract DNA is amber and copal inclusions that can embed organisms older than 30 millions of years (MY)^15,16^.

Fossilized tree resins represent an important research source, offering numerous opportunities to study biogeography, behavior, extinction and evolutionary changes over multiple generations. Arthropods, and especially insects, are the most abundant and well-preserved life forms in amber and copal, even if plants and rare soft-bodied vertebrates can also be maintained within fossilized resins^17–20^. Because amber and copal conserve three-dimensional form, color patterns and microscopic details of the engulfed organisms, such fossils can be easily compared with the relative extant specimens, representing a unique recapitulation of arthropods evolution starting from the Cretaceous era. Moreover, covering more than 200 MY of earth history and being spread throughout several climate zones, resin fossils provide an exceptional source of information to interpret past terrestrial environments and ecosystems^21–23^. Amber and copal are vegetal resins that have been fossilized, through a diagenetic process called amberization that is due to the terpenoid resins transformation^24^. The essential difference between amber and copal is their states of polymerization and their established ages^25^. Copal is usually considered an intermediate phase between fresh resin and amber, a completely fossilized resin that takes millions of years to transform from copal^26^. Resin, secreted from parenchymal plants cells in response to biotic or abiotic host trauma, can entrap living and dead organisms. Despite chemical composition of fossil resins exhibit extensive variations^27^, the complete and rapid engulfment of the organisms embedded seems to preserve specimen morphology and tissues structure during time. On the other hand, over millions of years of diagenetic events, required to transforms resin into copal before and amber subsequently, minimize the likelihood of aDNA preservation especially in amber specimens. Notably, when DNA is extracted from fossil inclusions, it can be used as a powerful tool to test new evolutionary hypotheses. In entomology, for example, ancient DNA can reveal new phylogenetic relationships between taxa, which are undetectable using other methodologies. However, the preservation of biomolecules in fossilized resins samples is still under debate when considering very old sample inclusions. Stankiewicz et al.^28^ detected chitin-protein complexes in insect cuticle from sub-fossil resins, while a recent study found amino acids from fossil feathers in amber from almost 100 million years BP^29^. Additionally, Bada et al.^30^ uncovered low levels of amino acid racemization rate in amber insect inclusions, implying that peptides could be preserved in these. Although there is no direct correlation between amino acid preservation and DNA survival^31^, theoretically, within this protective environment, DNA might be better conserved from external degradative processes compared to other substrates. On the other hand, because DNA degrades over time due to the spontaneous chemical breakdown^32,33^, it could be preserved in resins and young copals but almost certainly not in amber. Few studies have been performed in order to explore the possibility to extract uncontaminated DNA from copal and amber, yielding contrasting results^15,34–36^ ^(and references therein)^. Nevertheless, in 2013, Penney and colleagues^37^ were unable to obtain any convincing evidence for the preservation of endogenous aDNA in two copals with different age, dated to “post-Bomb” and 10,612 ± 62 cal. BP respectively, thus concluding that DNA is not preserved in this type of material, neither in the recent ones. On the contrary, molecules of endogenous DNA seem to be present in insects embedded in modern resins, albeit in small quantities. Recently, Peris et al.^38^ were able to amplify DNA from ambrosia beetles recovered in 6 and 2 year old resins, using a PCR-based method. Despite these positive results, access to ancient DNA molecules in older resins is still a challenge. In this work, we explored the possibility to obtain endogenous aDNA molecules from insects preserved in copal applying NGS technologies and experimental protocols specifically designed for recovering and analyzing highly degraded ancient DNA. This approach is more suitable than PCR-based method, allowing to distinguish ancient endogenous molecules from modern contaminants, as well as to evaluate DNA preservation status by fragmentation and deamination patterns analysis. The same procedures were successfully adopted to genotype very ancient specimens, such as Neanderthals, Denisova and *Homo heidelbergensis*^39–43^. Because the possibility of extracting insect aDNA from amber samples, dated millions of years ago, is still unknown^37^, we performed our analysis on two Colombian copal insect inclusions dated back 60 years. Moreover, aDNA isolation from insect inclusions requires a destructive sampling; for this reason, the relatively recent material we analyzed represent a suitable opportunity, in order to preserve rare and very old species stored in museum collections.

## Materials and methods

**Figure 1:**
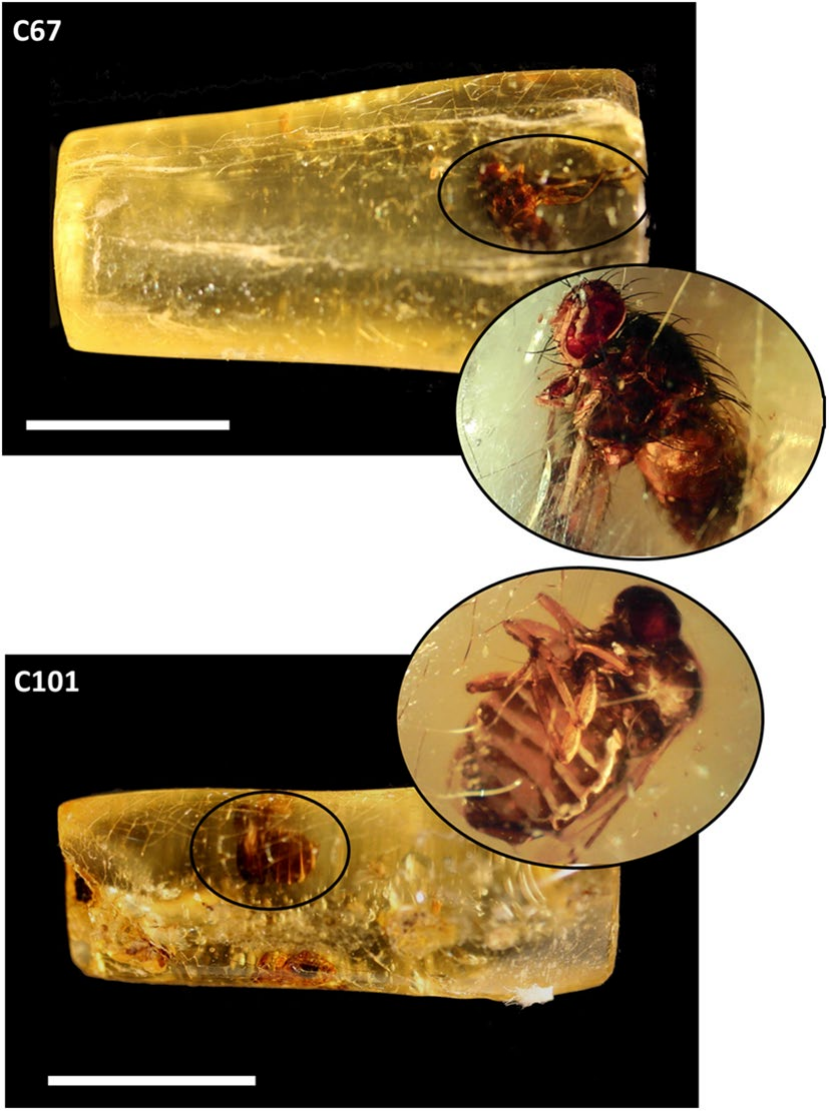
Analysed samples. Scale bar = 2 cm.

We analyzed two insects embedded in two Colombian sub-fossil resins, labeled C101 and C67 respectively (Fig. 1). Only one complete insect was present in each copal and no other equivalent specimens are available for further analyses. Copal were acquired by C.Z. for commercial use and the provided information regarding the regional location of the samples refer to Vélez—Santander (Colombia). To our knowledge, copals were untreated with high pressure heat to increase their durability and commercial appeal, which may have compromised the DNA preservation. C.Z. authorized sampling to conduct the scientific research. Direct radiocarbon dating was performed on each copal at the Beta Analytic testing Laboratory (London). Before sampling for molecular analysis, inclusions were morphologically characterized in order to identify their taxonomical attribution (Table 1).

**Table 1:** Samples description, morphological analysis results and radiocarbon dating results performed on resins.

Sample ID	Taxonomic attribution	Radiocarbon date		
		Fraction modern carbon	Conventional Radiocarbon Age	Calendar calibration
C101	Order, **Diptera** Suborder, **Brachycera** (probably Schizophora—Acalyptratae)	1.5480 ± 0.0058 (Beta—527,704)	− 3510 ± 30 BP	(95.4%) − 18 to − 21 cal BP
C67	Order, **Diptera** Suborder, **Brachycera** (probably Schizophora—Calyptratae)	1.6250 ± 0.0061 (Beta—527,706)	− 3900 ± 30 BP	(83.7%) (− 17 to − 18 cal BP)

Conventional Radiocarbon Ages and sigmas are rounded to the nearest 10 years per the conventions of the 1977 International Radiocarbon Conference. Because counting statistics has produced sigmas lower than ± 30 years, a conservative ± 30 BP is cited for the result, as reported by Beta Analytic.

Genetic analysis was performed in a dedicated aDNA facility, at the Laboratory of Molecular Anthropology and Paleogenetics (University of Florence, Italy), where no experiments have been previously performed on entomological samples. Preventive measures were taken to avoid contamination during all the experimental procedures^44,45^, and negative controls were processed during each step.

To remove environmental contaminants, copal was washed with 30% bleach and UV-irradiated (254 nm) for 30 min for each side. Sampling was carried out as described in Penney et al.^37^, with some modifications: each specimen was trimmed to a small cube using a micro-drill with disposable disk saw, taking care not to expose the insects. Inclusions were extracted dissolving, through rotation, the remaining resin in 5 mL chloroform for 24 h at RT conditions^46^, to minimize the DNA degradation risk. Each fully intact insect was drawn out from the dissolved copal and washed 3 times with 500 µL of Molecular Biology Grade water (ddH_2_O). Samples were finely crushed with a sterile micropestel and were subsequently incubated overnight at 55 °C in 1 mL of Digestion Buffer, which is specific for DNA extraction coming from chitinous tissues (10 mM Tris buffer pH 8.0; 10 mM NaCl; 5 mM CaCl_2_; 2 mM EDTA pH 8.0; 2% SDS; 25 mM DTT and 0.25 mg/ml Proteinase K)^7^. After pelleting, supernatant was extracted using a protocol properly designed for short fragments recovery^2^. DNA was quantified using QubitTM 4 Fluorometer (dsDNA High Sensitivity Kit). Twenty microliters of the extracts were used to prepare the libraries, following a custom double-indexing protocol optimized for ancient samples^47,48^. No uracil DNA glycosylase (UDG) treatment was performed in order to retain misincorporation profiles. Libraries concentration was determined with the Agilent 2100 Bioanalyzer (DNA 1000 chip). Afterwards libraries were pooled in equimolar amount with other samples and finally sequenced with an Illumina NovaSeq 6000 (SP flowcell) with a single-end 1 × 100 + 8 + 8 cycles at the Laboratorio di Genomica Avanzata, Univerity of Florence.

According to sample specific index sequences, raw reads were demultiplexed. EAGER pipeline^49^ was used for the initial reads quality control, the adapter trimming and for mapping the reads to the human reference genome in order to monitor and remove human contamination. FastQC^50^ was used to perform the quality control of the reads. Adapter sequences were trimmed with Clip&Merge v. 1.7.4^49^ and, fragments less than 30 base pairs (bp), were discarded. BWA v. 0.7.10^51^, set with aDNA specific parameters (-l 1000, -n 0.01, -o 2)^52^, was applied to map filtered reads to the human reference genome (Hg19, NCBI Build 38, December 2017). PCR duplicates were removed using DeDup^49^, and reads presenting a mapping quality lower than 30 (filtered with SAMtools-1.7^53^) were also omitted from the analysis. In order to identify the DNA sequences contained in our copal sample, we downloaded the entire NCBI database (version 4) (National Center for Biotechnology Information, www.ncbi.nlm.nih.gov) and built the related BLAST^54^ database files (version 2.9 database). Then we aligned our copal sequences previously processed with the de-novo assembler ABySS 2.0^55^ (parameters: -t 64 -pe openmpi 32) against the NCBI BLAST database. The xml BLAST results were parsed using a script written in Python 3 (https://github.com/tacclab/NCBIXML_parser) that used the module Bio.blast and the NCBIXML class in order to obtain a table including the sequence length, the taxonomic group, the corresponding database entry, the organism description, the length of the matching, BLAST Bit-score, E-value, the query start, the query end, the subject start and the subject end (See Supplementary Table S1). To validate our previous results we preprocessed the set of DNA sequences contained in our xml BLAST file with MEGAN6^56^. MEGAN computed and analyzed the taxonomical content of our dataset, employing the NCBI taxonomy to summarize and order the output.

The actual degradation of the embedded insects DNA was assessed by deamination and length patterns analysis. It has been shown that, over time, cytosines principally located at the ends of the fragments are deaminated to uracils^57^, which are sequenced as thymine residues. The C to T misincorporation patterns at the 5′-end of the molecule can be used to test the authenticity of aDNA sequences^58,59^. Since the complete genome of our identified insect is not available in the NCBI database, reads were aligned against rRNAs and mitochondrial genes related to identified the insect species *Ogcodes basalis* (See Supplementary Tables S1 and S2). Ancient DNA damage patterns were examined using MapDamage2^60^, setting –l 99 and –forward. As 100 bp single-end sequencing was carried out, mapDamage was performed on fragments up to 99 bp long in order to be sure about the full fragment sequence. Finally, cytochrome oxidase I (COI) sequence was called using mpileup and vcfutils.pl of the SAMtools-1.7 package^53^ and phylogenetically analyzed, to confirm the species-level identity of the copal insect inclusion. The software MUSCLE^61^ was used to align our COI sequence to that of six different *Ogcodes* species available in NCBI database (Supplementary Table S3). *Nemestrinus sp*. and *Stratiomyidae sp.* were used as out-groups. A phylogenetic tree was built with the Maximum Parsimony method (Subtree-Pruning-Regrafting algorithm) using MEGAX^62^. The tree was constructed with 99% partial deletion and phylogeny was tested with the bootstrap method with 1000 replications. Tree was then visualized by FigTree 1.4.4 (available from http://tree.bio.ed.ac.uk/software/figtree/).

## Results

We performed two direct radiocarbon analyses for our samples which both date to post-Bomb (Table 1). The initial qualitative and quantitative analysis showed differences in the DNA preservation degree between the two inclusions. DNA from C101 sample was 0.065 ng/µL, while DNA was not quantifiable for C67. Samples were converted into genomic libraries. After a first attempt starting from 20 µL of the extract, library was repeated for C67 starting from 30 µL of the same extracts. In both case, it was not possible to obtain a good quality library. According to these results, C67 was excluded from further analysis, thus C101 was successively sequenced. 33,271,931 reads were generated by shotgun sequencing (Table 2). Reads mapped to the human genome were 8220 (0.025%)–7430 (0.022%) after removing duplicates (Supplementary Table S4)—and they represent modern contamination, as shown by deamination profiles (Supplementary Fig. S1).

**Table 2:** Bioinformatics analysis results for *Ogcodes basalis*.

Sample ID	Number of raw reads	Mapped reads before DeDup	Mapped reads after DeDup	Cluster factor	CtoT (%)	Average fragment length (bp)
C101	33,271,931	277,876	9,845	27.1	21.93	88.96

Number of raw reads, number of mapped reads before and after PCR duplicates removal, cluster factor, damage at 5′ end, and average fragment length are reported.

**Figure 2:**
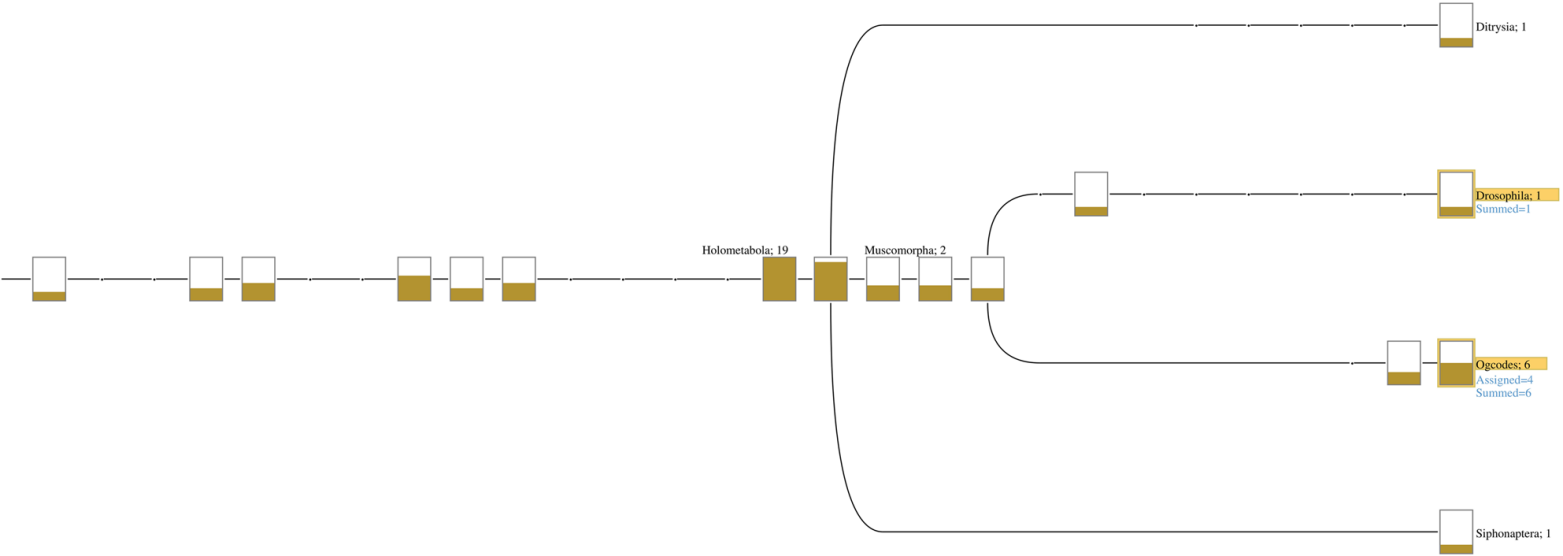
MEGAN6 results. Figure shows that the genus *Ogcodes* belong to the class with the highest assigned value.

Using ABySS 2.0, we assembled the non-human reads from the Fastq files, obtaining 18 assembled sequences with a length greater than 200. Seven sequences have also an E-value and a Bit-score less than 0.01 and greater than 394 respectively. Among the top seven sequences, the *Ogcodes basalis* species is the mostly identified organism by the BLAST algorithm (Supplementary Table S1), which was compared against all the genomes included in the NCBI repository. The vast majority of the hits associated to this species gave significant BLAST Bit-score higher than 190. Contigs showing lower scores have not been considered because they represent sequences phylogenetically associated to *Ogcodes* genus, such as the *Lysiphlebus testaceipes* wasp, the *Culicoides sonorensis* midge or the *Bactrocera cucurbitae* fly (see Supplementary Table S1). Same results were obtained using MEGAN6 (Fig. 2), where the genus *Ogcodes* belong to the class with the highest assigned value. We also found one hit matching with *Wolbachia sp*, with high Bit-score (298) and E-value less than 0.01 that showed typical features of aDNA (Supplementary Fig. S2).

Because *O. basalis* is the best represented taxon relative to other species of the genus in NCBI database, we performed a phylogenetic analysis among *Ogcodes* exemplars (Supplementary Table S3) based on COI sequence data, to confirm the attribution obtained with BLAST algorithm. We reconstructed the 64.98% of the COI with an average depth of coverage of 11.01. The phylogenetic position of the sample was assessed using Maximum Parsimony tree (Supplementary Fig. S3). C101 shows strong affinity with the *O. basalis*, confirming previous results.

Afterwards, raw reads were mapped against the genomic sequences, retrieved from NCBI database, of the *O. basalis* species (Supplementary Table S1). Despite incompleteness of the reference sequence, 277,876 mapped reads were obtained. However, after the duplicates removal, only 9845 reads were retained, showing a high value (27.1) of cluster factor (ratio of reads before and after PCR duplicate removal) (Table 2 and Supplementary Table S2). Since this measure is likely related to the library complexity level, high value of cluster factor indicates a low quality/quantity of the starting genomic material. The aforementioned value is significantly higher in the mapping statistic obtained for *O. basalis* than in the one gained for *Homo sapiens* (Supplementary Table S4). However, this result can be considered reasonable when looking at the different length of the analyzed genomes. Considering that the partial genome of the *O. basalis* used as reference has a length of 16,243 bp, the filtered reads obtained after duplicate removal are consistent to achieve a deep coverage (53.92%) of the entire sequence annotated. On the contrary, the number of reads mapping on the human reference genome are extremely low if compared to its extent. For this reason, experimental amplification is unlikely to have led to an over-representation of the initial human molecules, but it could have determined the high cluster factor for *O. basalis*. Comparable values of duplication rate were reached in Weyrich et al.^10^ where they compared the mapping statistics obtained for bacterial and human mitochondrial genomes which are characterized by a similar length.

**Figure 3:**
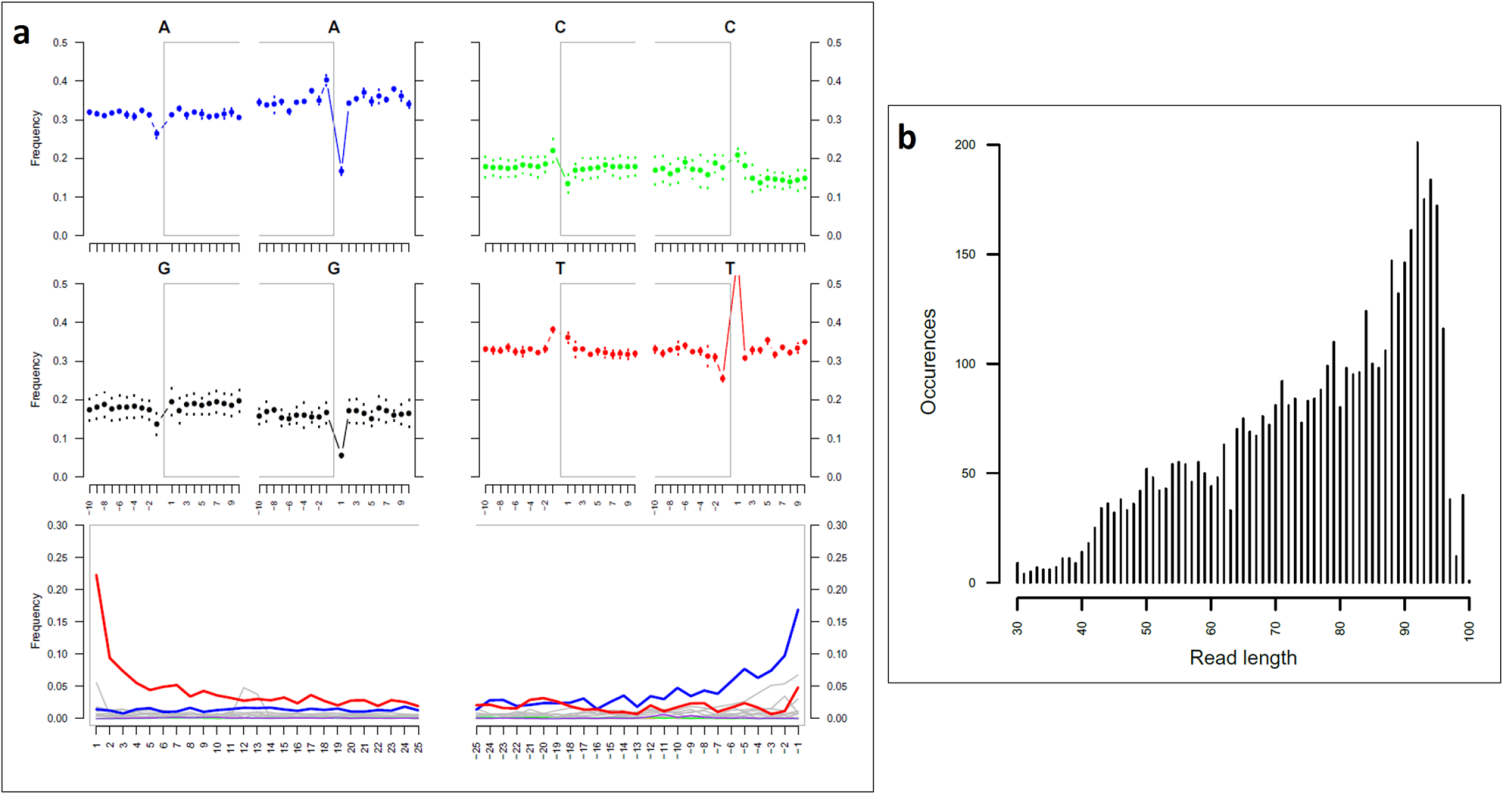
Deamination, base frequency and fragmentation patterns observed on *Ogcodes basalis*. Misincorporation plot (**a**, bottom plot) shows the percent frequency of C to T mismatches at 5′ read ends (in red print) and complementary G to A mismatches at the 3′ ends (in blue print); base composition plots (**a**, upper plots), describe the base frequency outside and in the read. Length distribution plot (**b**) show the frequency of read lengths.

Mapped reads displayed typical features of aDNA: short fragments length (average value of 88.96 bp), and high rate of C to T deamination (21.93%) at the 5′ termini of the molecules (Table 2 and Fig. 3), confirming the authenticity of data. Extraction and library negative controls yielded a total of 2,250,680 of reads, whereas only 56 of them mapped to the human genome. No reads associated to *O. basalis* or other fly insects were retrieved.

## Discussion

In this work, we applied the most innovative molecular and bioinformatics techniques in order to analyze two resin-entombed insects and their DNA preservation in Colombian copal. DNA was successfully extracted at very low concentration from sample C101 (0.065 ng/µL), while no genetic material was retrieved from the second one (C67). Despite the few amount of the starting genetic material, C101 sample yielded enough quantity of DNA to assess its preservation degree in the sub-fossil resin. One of the main issues in aDNA studies is contamination; DNA molecules from people who handle the samples can easily exceed the small amounts of endogenous aDNA^63–66^. In our case, the number of reads which mapped to the human genome, was very low, 8202 (0.025%) and 7430 (0.022%) before and after duplicates removal (Supplementary Table S4), suggesting that resin prevents and protects the inner environment and the endogenous DNA from external contaminants. BLAST alignment of our non-human assembled contigs, revealed significant percentages of similarity with *O. basalis* species (Supplementary Table S1). Similar results were obtained with MEGAN6^56^ where *Ogcodes* is the class with the highest assigned value (Fig. 2). Taxonomic attribution was also confirmed by phylogenetic analysis of the COI sequence. Maximum Parsimony tree shows clearly that C101 has a significantly higher affinity to *O. basalis* when compared to other *Ogcodes* exemplars (Supplementary Fig. S3). *Ogcodes* is the largest and widely distributed genus of the spider-parasite Acroceridae family, likely evolved during the late Triassic and greatly diversified during the Cretaceous^67^. Species belong to the genus show typical globose body, moderately small head and reduced wing venation. This group is relatively well represented in the fossil record, with some species being quite common in Baltic and Dominican amber deposits^68^. On the contrary, no *Ogcodes* exemplars are described in Colombian copal assemblages until now. Concordantly, same result was obtained with the observation of the phenotypic characteristics of the insect body embedded in the copal. This analysis identified our sample as belonging to the order *Diptera* and sub-order *Brachycera* that are the taxa to whom the genus *Ogcodes* belongs (Table 1). Interestingly, we also found one hit matching with *Wolbachia sp*, a gram-negative bacteria genus, that usually infects flying insects, especially species belonging to the Diptera order^69^. Because reads showed clear signatures of damage (Supplementary Fig. S2), it is reasonable to assume that this sequences arise from pathogens already present in the analyzed sample. Considering that typical microbial DNA molecules coming from environmental matrixes (e.g. soil) were not identified, our data can confirm that resins protect inclusions from external contamination. Although these are promising results, high proportion of low significance scores hits (see Supplementary Table S1) were detected. Probably, they are sequences derived from extremely degraded DNA. Indeed, when we performed a detailed analysis of the reads mapping *Ogcodes* genus, we found high degradation levels of the endogenous DNA (Table 2 and Fig. 3). First, the high value of cluster factor (27.1) indicates a low complexity levels in the sequenced library. Since this parameter is correlated with the quality and the quantity of the starting DNA^70^, these results lead us to question about the preservation of the endogenous genetic material. Another possible explanation for the high value of cluster factor is the suboptimal procedure applied to release the embedded insect. Indeed, Peris et al.^38^ found that chloroform used for dissolving resin surrounding the specimen could compromise DNA concentration. Secondly, damage pattern observed at the ends of the fragments (21.93% at 5′ end) highlights a high level of degradation (Fig. 3). Sawyer et al.^59^ reported a strong positive correlation between C to T substitutions and the age of the samples for bone remains; for this reason, nucleotide misincorporation patterns can be used as a valuable criteria to distinguish recent DNA sources from ancient ones. However, given the young age of the copal, molecules from *O. basalis* showed C to T frequencies higher than other samples with the same age range^59^. Indeed, despite their age, while C67 sample did not provide any genetic material, endogenous DNA extracted from C101 was low concentrated and extremely degraded.

## Conclusion

Resin fossils represent a unique palaeobiological resource. Given their peculiar fossilization process and the exceptional morphological and biochemical preservation, they were thought to be the best means to obtain genetic material from ancient specimens. Although previous studies claim the impossibility to recover endogenous DNA from copal and amber, our positive results demonstrate that also sub-fossil resin inclusions, even though with cautions, may be useful in aDNA researches. Indeed, we were able to identify the taxonomic status of the specimen embedded in a Colombian copal following the most innovative molecular techniques developed to analyze highly degraded DNA. At the same time, we ascertained and described a high level of DNA degradation, despite the young age of the specimen. For this reason, because of the destructive method of DNA extraction protocols and the non-reproducibility of the results, we strongly discourage aDNA analysis from amber-preserved fossils, even if our study was carried out on copal inclusion.

## Supplementary Information

Supplementary Table 1.

Supplementary Information.

## Data Availability

Raw data are available at the NCBI Sequence Read Archive under Accession Number PRJNA633577.
