# Supplementary material for: Successful extraction of insect DNA from recent copal inclusions: limits and perspectives
Source: Sci Rep. 2021 Mar 25;11:6851. doi: 10.1038/s41598-021-86058-9 (PMC7994385; doi:10.1038/s41598-021-86058-9)
Supplement: Supplementary file 2 — Supplementary Information. [file 41598_2021_86058_MOESM2_ESM.pdf]

Successful extraction of insect DNA from recent copal inclusions: limits and perspectives

Alessandra Modi[1*†], Chiara Vergata[1†], Cristina Zilli[2], Chiara Vischioni[3], Stefania Vai[1], Guidantonio Malagoli Tagliazucchi[4] Martina Lari[1], David Caramelli[1], Cristian Taccioli[3*]

[1] Department of Biology, University of Florence, 50122 Florence, Italy

[2] Indipendent researcher, Padova, Italy

[3] Department of Animal Medicine, Production and Health, University of Padova, 35020 Legnaro (PD), Italy

[4] UCL Genetics Institute, Department of Genetics, Evolution and Environment, University College London, Darwin Building, Gower Street, London WC1E 6BT

[†]these authors contributed equally to the manuscript

SUPPLEMENTARY TABLES

Table S2. ***Ogcodes basalis* genes**. List of *O. basalis* genes used for mapping, accession ID, sequences length and mapped reads on each gene are reported.

Definition [Accession]	Sequence length (bp)	Reference	Number of mapped reads
**6-phosphogluconate dehydrogenase (PGD) gene [KC177498]**	732 (partial cds)	[1]	5
**18S ribosomal RNA gene [KC177292]**	2011 (partial sequence)	[1]	3,532
**28S ribosomal RNA gene [KC177696]**	3832 (partial sequence)	[1]	5,558
**alanyl-tRNA synthetase-like protein (AATS) gene [KC177615]**	1701 (partial cds)	[1]	11
**C1-THF synthase-like protein (PUG) gene [KC177539]**	294 (partial cds)	[1]	4
**Glucose-6-phosphate 1-dehydrogenase (G6PD) gene [KC178087]**	744 (partial cds)	[1]	3
**Sans fille (snf) gene [KC177064]**	264 (partial cds)	[1]	1
**Seven in absentia (sina) gene [KC178338]**	441 (partial cds)	[1]	15
**Syntaxin 1A (syx1A) gene [KC177840]**	400 (partial cds)	[1]	4
**Triosephosphate isomerase (tpi) mRNA [KC177926]**	474 (partial cds)	[1]	1
**16S ribosomal RNA gene [AY140867]**	529 (partial sequence)	[2]	521
**Cytochrome oxidase subunit I (COI) gene [DQ631991]**	731 (partial cds)	[2]	158
**Carbamoyl-phosphate synthase gene [AF539886]**	871 (partial cds)	[2]	0
**Carbamoyl phosphate synthetase-aspartate transcarbamoylase-dihydroorotase gene [HM183063]**	3219 (partial cds)	[3]	32

Table S3. COI sequences used in phylogenetic analysis. *Ogcodes* species and accession ID are reported.

Species	Accession ID
O. basalis	DQ631991
O. dispar	MN411178
O. egonatus	MF637865
O. fumatus	HE575183
O. canadensis	DQ631975
O. reginae	HF955418
Nemestrinus sp.	MG968022
Stratiomyidae sp	JF872312

Table S4. **Bioinformatics analysis results for human genome**. Number of mapped reads on human genome before and after PCR duplicates removal, mapped reads against human mitochondrial genome, percentage of human DNA, damage at 5' end, and average fragment length are reported.

Sample ID	Mapped reads before DeDup	Mapped reads after DeDup	Mapped reads on mitochondrion	Human DNA (%)	C to T (%)	Average fragment length
**C101**	8202	7430	4	0.022	3.6	72.58

SUPPLEMENTARY FIGURES

Figure S1. Deamination (A) and fragmentation (B) patterns observed on *Homo sapiens* reveal a set of undamaged sequences deriving from modern contaminants.

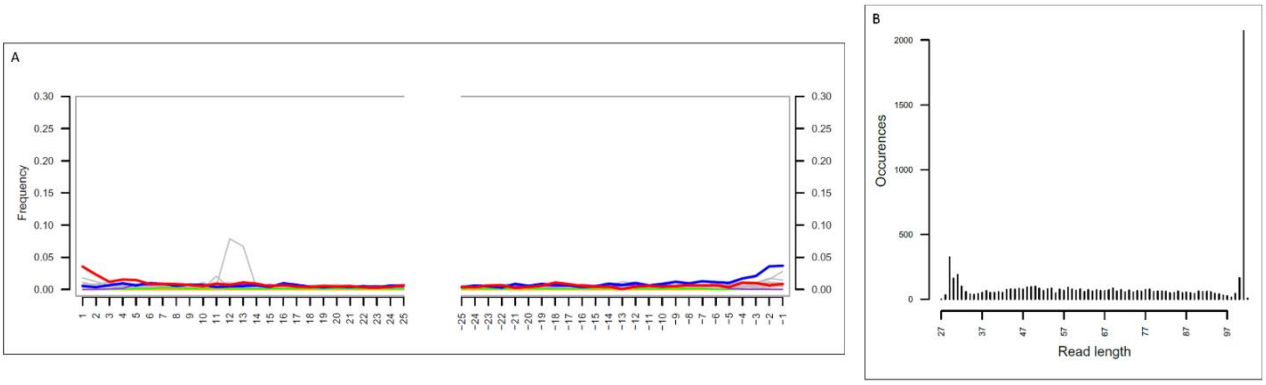

Figure S2. **Deamination (A) and fragmentation (B) patterns observed on *Wolbachia sp***

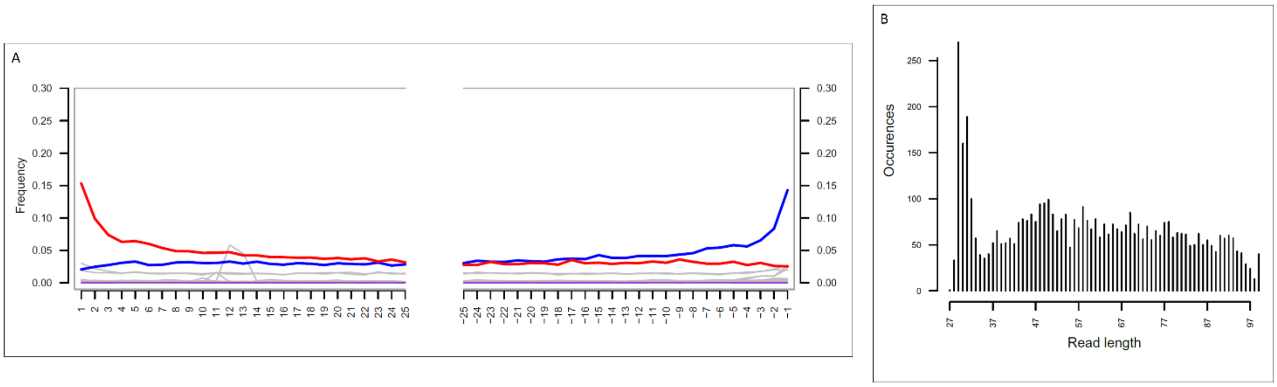

Figure S3. Maximum Parsimony tree constructed for C101 and 6 published COI of *Ogcodes* species. C101 are lebelled in red. *Nemestrinus sp*. and *Stratiomyidae sp*. were used as phylogenetic out-groups. Nodes with posterior probability > 0.5 are reported.

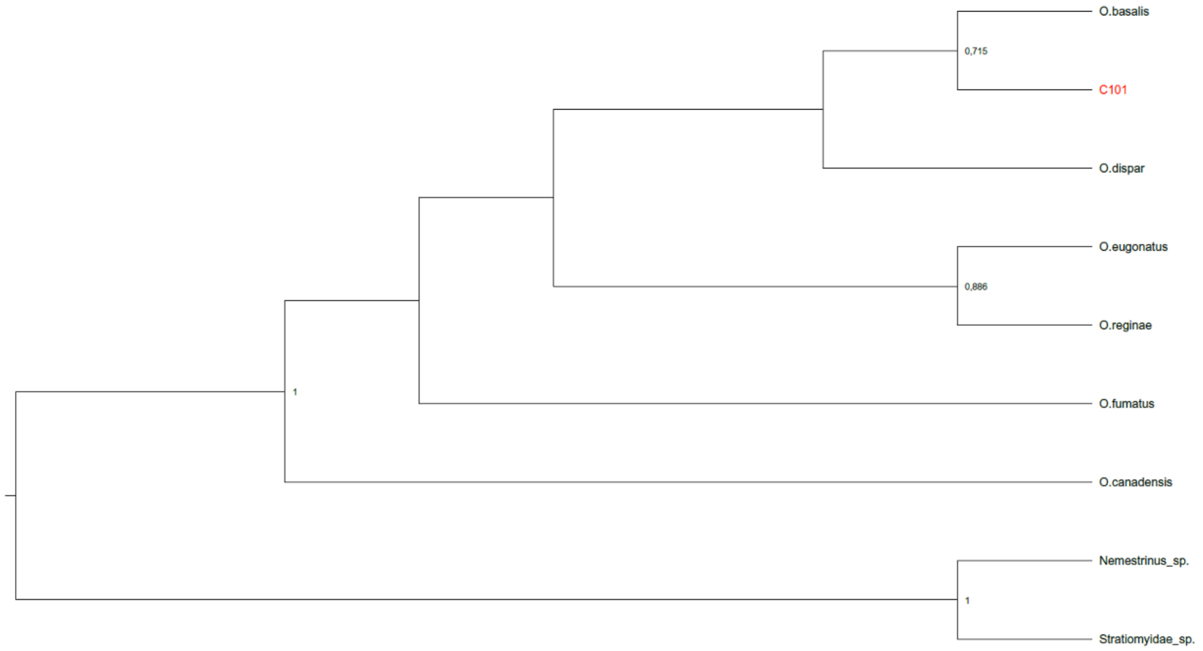
O.basalis
0,715
C101
O.dispar
O.eugonatus
0,886
O.reginae
1
O.fumatus
O.canadensis
Nemestrinus_sp.
1
Stratiomyidae_sp.

References

1. Wiegmann, B.M. et al. Episodic radiations in the fly tree of life. *Proc. Natl. Acad. Sci. U S A* **108**, 5690-5695 (2011)

2. Winterton, S.L. et al. Phylogeny and Bayesian divergence time estimations of small-headed flies (Diptera: Acroceridae) using multiple molecular markers. *Mol. Phylogenet. Evol*. **43**, 808-832 (2007)

3. Trautwein, M.D. et al. A multigene phylogeny of the fly superfamily Asiloidea (Insecta): Taxon sampling and additional genes reveal the sister-group to all higher flies (Cyclorrhapha). *Mol. Phylogenet. Evol*. **56**, 918-930 (2010)
